# Inverse Fusion PCR Cloning

**DOI:** 10.1371/journal.pone.0035407

**Published:** 2012-04-17

**Authors:** Markus Spiliotis

**Affiliations:** Institut für Parasitologie, Universität Bern, Bern, Switzerland; Mayo Clinic, United States of America

## Abstract

Inverse fusion PCR cloning (IFPC) is an easy, PCR based three-step cloning method that allows the seamless and directional insertion of PCR products into virtually all plasmids, this with a free choice of the insertion site. The PCR-derived inserts contain a vector-complementary 5′-end that allows a fusion with the vector by an overlap extension PCR, and the resulting amplified insert-vector fusions are then circularized by ligation prior transformation. A minimal amount of starting material is needed and experimental steps are reduced. Untreated circular plasmid, or alternatively bacteria containing the plasmid, can be used as templates for the insertion, and clean-up of the insert fragment is not urgently required. The whole cloning procedure can be performed within a minimal hands-on time and results in the generation of hundreds to ten-thousands of positive colonies, with a minimal background.

## Introduction

Commercially available systems allowing the directional insertion of PCR products into vectors such as the topoisomerase based Champion system (Invitrogen) are easy to use but limited to the vectors provided and to fixed cloning sites. Alternative methods for insertion of PCR products, allowing a free choice of the insertion site have been developed. These methods can be divided in two groups, namely systems where additional sequences such as restriction or recombination sites are present at the insertion-boundaries after cloning [1]–[3] and seamless cloning methods without additional sequences [4]. In a directional way, seamless cloning of PCR products into linearized vectors can be performed by applying type-II restriction enzymes [5]. Despite their differences in implementation, other seamless cloning methods start with a linearized vector and a PCR amplified insert containing sequences that are homologous to the vector on both ends. With this starting material seamless cloning can be performed by *in vivo* recombination using special *E. coli* strains [6], by *in vitro* recombination using *E. coli* extracts [7] or by annealing, if long single stranded cohesive ends are present in the vector and insert sequences. These cohesive ends can remain as a result of incomplete PCR after vector and insert amplification [8], [9], they can be generated in a PCR setup [10] or prepared enzymatically [8], [11]. The commercially available systems In-Fusion (Clontech), Geneart (Invitrogen) or CloneEZ (GenScript) are based on enzymatically prepared cohesive ends. Another possibility to combine vector and insert is to fuse both in a non-exponential PCR setup, which can be performed with linearized [12] or circular vector [13]–[15]. Most of the alluded methods result in insert-vector fragments containing gaps or nicks that are filled and ligated by the cellular DNA repair machinery after transformation. Commonly, the insert is amplified by PCR and with the exception of the last PCR based setups, the vector is prepared by restriction digestion, or alternatively by PCR in case no adequate restriction sites are present. Depending on the cloning method, more than 200 ng linear vector DNA per reaction is necessary, and quantitative preparation of the high molecular vectors by PCR is, however, not very effective. Additionally, plasmids might not be efficiently digested, which may lead to false positive background due to uncut vector. Next to the large amounts of insert and vector DNA, some of the methods require additional complex or time consuming working steps, many and/or long primers, or they have a low yield in colony numbers. As a consequence, seamless directional cloning could be optimized by (i) reducing the number of steps required for the preparation of insert and vector, (ii) reducing the amount of required starting material, and (iii) increasing the yield of positive colonies.

**Figure 1:**
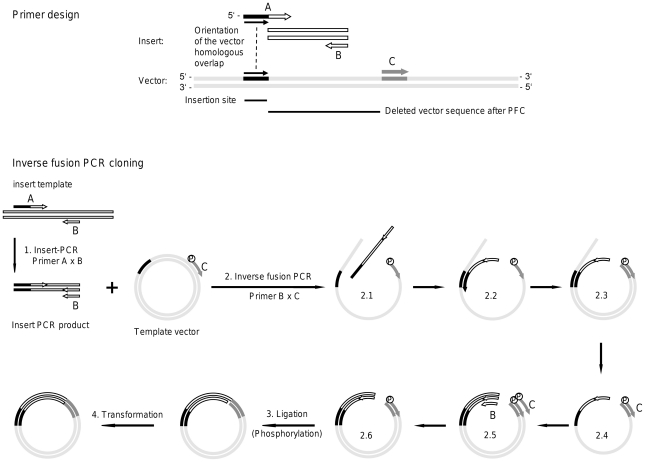
Schematic outline of inverse fusion PCR cloning (IFPC). (Primer design) 3 primers are required for IFPC. For the amplification of the insert, the forward primer A and the reverse primer B are used. Primer B is an insert-specific standard primer while the 5′-end of primer A is comprised of a sequence homologous to the desired insertion site of the vector (black) and the 3′-end is specific for the insert (white). The annealing site for the vector primer C has to be chosen downstream of the insertion site, and must not overlap with the insertion site. The annealing Tm of primer B, primer C, the vector homologous part of primer A as well as the insert specific part of primer A should all be around 58°C. Depending on how IFPC will be performed, primer B or primer C can be 5′-phosphorylated (see below). The sequence between the insertion site and primer C will be deleted after IFPC. **(Inverse fusion PCR cloning)** (1.) The insert (white) is amplified via primers A and B and should be gel-eluted when unspecific PCR products or smears appear. (2.) For the inverse fusion PCR, a mix containing insert-PCR product, circular plasmid template, primer B and phosphorylated primer C is prepared. In the first rounds of PCR, forward strands of vector and reverse strands of insert are enriched by primer-extension of primers B and C in a linear way (2.1). Then, the insert reverse strands anneal with their vector homologous 3′-end to the complementary sequence (black) of the linear plasmid forward strands (2.2.) and the inserts are elongated by overlap extension (2.3.), thus forming the fused insert-plasmid template lacking the original sequence of the template plasmid between the insertion site and primer C (2.4.). The second strand of the template is generated by primer extension of primer C, finalizing the double-stranded template (2.5.), which is now exponentially amplified via primer B and C (2.6.). The linear insert-plasmid fusions are now circularized by T4-ligation (3.). As an alternative to phosphorylated primer C, a phosphorylated primer B can be used, or the phosphorylation can be incorporated by T4-polynucleotide-kinase treatment during the ligation step. Finally the ligated insert-vector fusions are transformed into competent *E.coli* (4.), where the bacterial DNA repair machinery will close the nick of the second strand. A working protocol is shown in material and methods.

Inverse fusion PCR cloning (IFPC) as it is described here fulfils all three criteria. IFPC requires no preparative work to be done on the vector DNA, and only one facultative clean up step for the insert DNA. Moreover, very low amounts of starting material are necessary, and high counts of positive colonies are achieved, with a minimal background. The principle of IFPC is schematically drawn in Figure 1. IFPC is a combination of two established PCR methods, namely a fusion- or overlap extension-PCR [16] which allows the joining of two PCR products, and an inverse-PCR [17], [18], which allows e.g. the insertion of point mutations into plasmids or the deletion of plasmid sequences. A combination of both PCR methods, named here inverse fusion PCR, is used for directional seamless insertion of PCR products containing a vector-complementary 5′-end into plasmids, with a free choice of the insertion site. An important feature of IFPC is the exponential amplification of the fused vector-insert fragments during the inverse fusion PCR step. Thus minimal amounts of starting material (>0.5 ng for a 4 kb plasmid and ∼10 ng for a 1 kb insert) can be used to produce high yields of colonies. Only three primers are needed to perform IFPC. The insert is amplified with the forward primer A and the reverse primer B, and primer A contains a 5′-sequence homologous to the desired vector insertion site. For the inverse fusion PCR step, insert, vector, primer B and the vector specific primer C are used. During inverse fusion PCR two things happen. First the reverse insert strand anneals to the complementary vector sequence with its vector homologous 3′-end like a primer and is then elongated by primer extension, thus forming the fused insert-vector template. In a second step the linear insert-vector fusion is exponentially amplified via primers B and C. For the inverse fusion PCR a high fidelity DNA polymerase with proofreading activity should be used to minimize PCR artefacts and to produce blunt ended fragments. These can be circularized by ligation. For the ligation at minimum one of the 5′-ends of the insert-vector fragment has to be phosphorylated. This can be achieved by using either a 5′-phosphorylated primer B or primer C in the inverse fusion PCR step or by phosphorylating the final insert-vector fragments with T4-polynucleotide kinase (pnk). When a phosphorylated primer is used, the final circularized fragments contain a nick at the 5′-end of the non phosphorylated primer that will be closed by the cellular DNA repair machinery after transformation. The hands-on time for performing IFPC is very low, since bacteria containing the vector and diluted insert PCR can be used as templates for the inverse fusion PCR, which is then just mixed with ligation buffer and ligase prior transformation. If non phosphorylated primers were used, additionally pnk has to be added to the ligation mix.

## Results

### Experimental setup (Figure 2 A)

**Figure 2:**
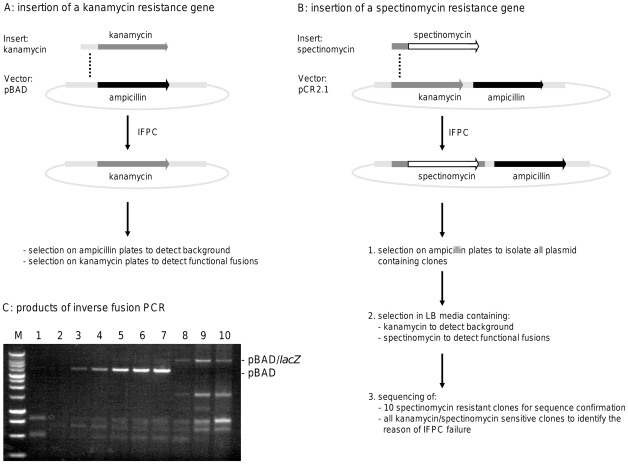
Experimental setup for the exchange of antibiotic resistances by inverse fusion PCR cloning. (**A**) The ampicillin resistance gene of the vector pBAD was exchanged by a kanamycin resistance gene. Via selection on ampicillin plates the residual background was detected, and through kanamycin selection the functional fusions were identified. This setup positively selected functional fusions and was used to optimize the system. (**B**) The kanamycin gene of the vector pCR2.1 was exchanged by an in-frame insertion of a spectinomycin gene (*aadA*). (1) In a first round, the ampicillin resistant colonies were selected, containing the vector with or without insertion. (2) In a second round, 192 of the ampicillin resistant colonies were used. Background containing the original vector was selected in LB media by addition of kanamycin and functional *spectinomycin* fusions by addition of spectinomycin. (3) 10 spectinomycin resistant clones were sequenced for sequence confirmation and all clones sensitive to kanamycin and spectinomycin were sequenced to identify the reason of IFPC failure. With this setup the insertion of non selectable sequences could be mimicked, because background (kan^R^), successful insertions (spec^R^) and failed fusions (kan^S^/spec^S^) were detected. By calculating the relationship between successful insertions and failed fusions, the failure frequency of IFPC was specified to be 6.5% in this experiment. (**C**) Results of an inverse fusion PCR to change the ampicillin into a kanamycin resistance in the vectors pBAD (lanes 1–7) or pBAD-TOPO/*lacZ*/V5-His (lanes 8–10). The molarity of vector and insert templates in lanes 1–7 correspond to the amounts shown in Table 1 (N°. 1–7) and in lanes 8–10 to the amount shown in Table 1 (N°. 7), but 25 cycles (lane 8), 30 cycles (lane 9) and 35 cycles (lane 10), respectively, were performed in the inverse fusion PCR step. The PCR was run using primer B1 and primer C1-b. 10 µl of each PCR were loaded on the gel and resulted after ligation and transformation in 260.000 cfu (lane 7), 12.000 cfu (lane 8), 19.600 cfu (lane 9) and 15.700 cfu (lane 10) per µl of inverse fusion PCR. M: Bench Top1 kb ladder (Promega).

In order to characterize IFPC as a method for functional expression of proteins in *E. coli*, the replacement of the ampicillin resistance gene of the vector pBAD by a kanamycin resistance gene was chosen, thus allowing a simple screen for functional insertions by counting kanamycin resistant colonies versus ampicillin resistant background colonies. Clean-up or enzymatic treatment steps were reduced by using circular vector as template for the inverse fusion PCR and a T4-ligation system working with crude PCR reactions. Since some settings resulted in very high counts of positive clones, the number of colonies is given as a value corresponding to 1 µl of the initial inverse fusion PCR and not as the commonly used clone forming units (cfu) per µg of DNA. The first PCR (amplification of insert) was performed under standard conditions. In order to optimize the second or inverse fusion PCR conditions, three main parameters were tested. These were (i) the amount of circular template vector pBAD (1 pg to 50 ng), (ii) the ratio of vector to insert (1: 0.1 up to 1: 1000), and (iii) the quality of the insert (crude PCR, column-cleaned PCR, or the gel-eluted insert). Moreover, *E. coli* containing the vector pBAD were used as a template for the inverse fusion PCR. Finally the 5′-phosphorylation of the fusion PCR products by a T4-polynucleotide-kinase was compared to the use of phosphorylated primers.

### Evaluation of IFPC

A good range for the amount of vector to be used was between 12 and 20 pg per 1 µl of inverse fusion PCR, yielding between 10,700 and 24,000 colonies per µl fusion PCR. Employing lower amounts resulted in reduced colony numbers (Table 1, N°. 1–7), and higher amounts (>100 pg µl^−1^) yielded high numbers of residual ampicillin resistant background colonies (data not shown). The ideal vector to insert ratio was 1∶100. Higher ratios did not result in more colonies (data not shown) and lower ratios again resulted in fewer colonies (Table 1, N°. 7–10). The quality, or purity, of the insert DNA had a clear influence on the numbers of positive colonies. Gel-purified insert DNA gave the highest number of colonies, namely 24,000, while for column-cleaned insert DNA, the yield was reduced to 50%, and with diluted crude PCR products, still 6600 cfu per µl inverse fusion PCR reaction were obtained, corresponding to a 75% reduction as compared to gel-purified insert DNA (Table 1, N°. 7 as compared to 11 and 12). All these inverse fusion PCRs were performed with a long annealing step starting from 65°C followed by slowly decreasing the temperature at 0.1°C s^−1^ down to 58°C. To speed up the inverse fusion PCR, the annealing step was shortened to 30 s at 58°C, which resulted in a decrease of colony numbers of only 20% (Table 1, N°. 7 as compared to 13). Since with crude product of the insert PCR the number of kanamycin resistant colonies was still around 6,600 per µl of the inverse fusion PCR reaction, it was tempting to test whether the whole procedure would succeed without any cleanup step. Thus, the inverse fusion PCR was performed with an *E. coli* colony harbouring pBAD, suspended in H_2_O and mixed with diluted crude insert PCR. The amount of kanamycin resistant colonies was clearly reduced as compared to the use of purified plasmid and insert, but depending on the dilution, up to 830 colonies could still be detected (Table 1, N°. 14–18 as compared to 11).

**Table 1:** Conditions and cloning rates for *kanamycin* insertion into pBAD by IFPC^a^.

N°.	Vector (V) and Insert (I) per 1 µl of inverse fusion PCR setup	Molarity V: I (pM)	Ratio V: I	Kan^R^ cfu µl^−1^ fusion PCR	Amp^R^ cfu µl^−1^ fusion PCR	variable conditions^b^
1	0.2 pg V+4 pg I	0.08: 8.0	1∶100	-	-	
2	2 pg V+40 pg I	0.8: 80	1∶100	12	-	
3	4 pg V+80 pg I	1.6: 160	1∶100	600	-	
4	8 pg V+160 pg I	3.2: 320	1∶100	1,100	2	
5	12 pg V+240 pg I	4.8: 480	1∶100	10,700	7	
6	16 pg V+320 pg I	6.4: 640	1∶100	23,000	4	
7	20 pg V+400 pg I	8.0: 800	1∶100	24,000	1	
8	20 pg V+200 pg I	8.0: 400	1∶50	6,900	4	
9	20 pg V+40 pg I	8.0: 80	1∶10	200	3	
10	20 pg V+4 pg I	8.0: 8.0	1∶1	20	4	
11	20 pg V+400 pg I	8.0: 800	1∶100	6,600	4	insert: diluted crude PCR
12	20 pg V+400 pg I	8.0: 800	1∶100	11,800	8	insert: PCR column cleanup
13	20 pg V+400 pg I	8.0: 800	1∶100	19,500	9	annealing: 30 s – 58°C
14	1 colony^c^ in 50 µl H_2_O+400 pg I	n.d.: 800	-	-	13	
15	1 colony^c^ in 500 µl H_2_O+400 pg I	n.d.: 800	-	420	14	
16	1 colony^c^ in 5 ml H_2_O+400 pg I	n.d.: 800	-	830	6	
17	1 colony^c^ in 50 ml H_2_O+400 pg I	n.d.: 800	-	60	-	
18	1 colony^c^ in 500 ml H_2_O+400 pg I	n.d.: 800	-	19	-	
19	20 pg V+400 pg I	8.0: 800	1∶100	3,400	-	primers B2×C2
20	20 pg V+400 pg I	8.0: 800	1∶100	1,540^d^	3	primers B1×C2, T4-pnk
21	20 pg V+400 pg I	8.0: 800	1∶100	1,870 (9,350)^d^	1	primers B1×C2, T4-pnk, 0.2 µl inverse fusion PCR
22	4 ng I	-: 8,000		-	-	control: insert alone
23	20 pg V	8.0: -		-	39	control: vector alone

a) For comparability all data shown was generated in one parallel setup.

b) The standard procedure and PCR conditions are described in the material and methods part. If not other mentioned, gel-eluted insert and plasmid derived from a mini-prep were used as templates. Normally only a very light or even no band is visible on an agarose gel when 10 µl of the inverse fusion PCR was loaded.

c) Instead of plasmid an *E. coli* colony containing pBAD was used. pBAD is a high copy plasmid. Low copy plasmids will need lower dilutions for optimal IFPC performance.

d) Since the (NH_4_)SO_4_ present in the PCR buffer inhibits phosphorylation by T4-pnk, one experiment was performed with 2 µl fusion PCR while the other one was prepared with 0.2 µl. 1,870 colonies were counted per 0.2 µl of fusion PCR, for comparison 9,350 colonies are the calculated colonies per µl of fusion PCR.

Subsequent investigations were done to see whether phosphorylation of primer B versus primer C influences the yield of positive colonies, and whether there is an effect in case the inverse fusion PCR product is phosphorylated with polynucleotide kinase (T4-pnk) during a reaction employing non-phosphorylated primers. With a phosphorylated primer C, the number of positive colonies was 7 times higher than with a phosphorylated primer B (Table 1, N°. 7 as compared to 19). Compared to a control with a phosphorylated primer C nearly 15 times less colonies were obtained when a non-phosphorylated primer C was used and the inverse fusion PCR products were treated with T4-polynucleotid kinase in one step in the ligation mix (Table 1, N°. 7 as compared to 20–21).

Finally, two different ligation systems working in the presence of PCR reaction buffer were tested, namely the quick ligation system from NEB and the rapid ligation system from Promega. Both systems resulted in almost similar counts of colonies. In contrast, isopropanol-precipitation of the fused PCR products followed by a standard T4-ligation resulted in less than 10% colonies (data not shown). Since the ligation is normally carried out at room temperature and the pnk-phosphorylation is done at 37°C, both setups were also incubated at 37°C resulting in very low counts of colonies (data not shown).

### Error frequency of IFPC (Figure 2B)

The experiments described above were dealing with the number of positive, thus functional, fusions selected by the exchange of the ampicillin into a kanamycin resistance. In the next step, the numbers of negative, non-functional fusions were determined. With this setup the insertion and the expected error rate of non selectable sequences can be mimicked. For this, the kanamycin resistance gene of the vector pCR2.1 (both, amp^R^ and kana^R^) was exchanged by the in-frame insertion of a spectinomycin resistance gene to detect non-functional fusions. The typical read-outs of this experiment were ampicillin and spectinomycin - but not kanamycin - resistance in the case of a functional fusion, or only ampicillin resistance in the case of a non-functional fusion due to deletion of the kanamycin resistance gene by insertion of a non-functional spectinomycin resistance gene. From 192 randomly picked ampicillin resistant colonies 172 (∼90%) were ampicillin and spectinomycin resistant, representing functional fusion events. 96 of these functional colonies were tested by a specific colony PCR resulting in an identical banding pattern for all colonies (data not shown). Furthermore, plasmids of 10 of these colonies were sequenced and no alterations were detected on the 5′- and 3′-boundaries or inside the spectinomycin sequence. Seven of the ampicillin resistant colonies (∼3.5%) still contained a functional kanamycin resistance gene, indicative for background. 13 of the ampicillin resistant colonies (∼6.5%) were susceptible to spectinomycin and to kanamycin and thus represented non-functional fusion events. These colonies were analyzed by sequencing to detect the reason for the failure of functional fusion. One colony contained no spectinomycin sequence but a mutated non-functional kanamycin resistance gene. Six colonies were found to be fusions of the template vector pCR2.1 and different bacterial DNA sequences and six colonies exhibited truncated spectinomycin sequences lacking parts of their C-terminus due to mismatched primer B3.

### Optimization of IFPC

With similar settings, the pCR2.1-spectinomycin experiment resulted in 1430 positive colonies, representing only ∼5% of the yield obtained in the pBAD-kanamycin experiment (data not shown). In rare cases similar reduced clone counts were observed for single inserts when different inserts were cloned using the same vector insertion site (data not shown). Additionally, less than 100 positive colonies were observed when the ampicillin/kanamycin resistance exchange approach was performed with the vector pBAD-TOPO/*lacZ*/V5-His. Since the only difference between pBAD used for the optimization experiments and pBAD-TOPO/*lacZ*/V5-His is the presence of the full *lacZ* gene in the latter one, it was mentioned that the low colony yield was a result of poor template amplification. To address this question, two experimental setups were performed with the vector pBAD-TOPO/*lacZ*/V5-His. First the cycle number of the inverse fusion PCR was increased and as a result 30 cycles yielded in 920 colonies and 35 cycles in 1560 colonies. Because primer C1 has a very low melting temperature of ∼50°C, a second IFPC was performed with primer C1-b (Tm 62°C), yielding 12,000 colonies after 25 cycles, 19,600 colonies at 30 cycles, and 15,700 colonies at 35 cycles. The control setup using pBAD as template and C1-b as a primer resulted in 270,000 colonies per µl of inverse fusion PCR. At 25 cycles the yield of colonies was approximately 5% compared to the control. 60% more colonies were obtained by adding 5 cycles to the inverse fusion PCR. The final inverse fusion PCR products (10 µl per lane) are shown in Figure 2 C. It should be noticed at this point, that these PCR products were prepared with primer C1-b and that for almost all experiments described before (pBAD/optimization, pCR2.1/spectinomycin) only very light or even no bands were visible in the gel (data not shown), but nevertheless resulted in high colony counts.

## Discussion

IFPC is a fast cloning method, requires low amounts of starting material, few experimental steps, and results in a high yield of positive colonies. To perform an IFPC, a bacterial colony containing the template vector can be suspended in a convenient amount of water, and an inverse fusion PCR reaction is performed with the desired insert. This procedure already gives high yields of positive colonies but by using a purified vector template and a gel-eluted insert the yield can be increased by two orders of magnitude. IFPC tolerates a wide range of setup possibilities and is therefore successful even under non-ideal conditions, where unfavourable vector to insert ratios or very low amounts of template are found.

In both described setups the vector homologous overlap of primer A was calculated with an annealing Tm of around 58°C, but overlaps with lower annealing Tms did also provide good results (data not shown). If the insert PCR results as a clear band without additional products, it can be used as a diluted PCR, but if smears or additional products are present, a gel elution is recommended. A high-fidelity DNA polymerase with proofreading activity should be used, since blunt ends are mandatory for the final ligation and enzymes such as Phusion (FinnZymes or NEB) show a very low failure frequency. The 5′-phosphorylation necessary for the ligation can be incorporated by 5′-phosphorylated primers B or C resulting in high number of colonies, or by phosphorylation with T4-polynucleotide-kinase resulting in a lower yield of colonies most likely due to the presence of ammonium sulphate in the PCR buffer and a suboptimal reaction temperature of 25°C. The amount of inverse fusion PCR used for the combined phosphorylation/ligation step still has place for optimization, but it results in the described settings in sufficient colony counts, thus saving time by avoiding additional cleanup steps or other experimental treatments. Combined phosphorylation/ligation may be the method of choice in the case of many IFPCs on different insertion sites or template vectors or in the case phosphorylated primers are too expensive. An important factor for the high yield of colonies as described here is the usage of highly competent *E.coli*. Therefore, a meaningful comparison between different cloning methods as described in the introduction is not really possible. Normally nobody requires 20,000 identical clones, but highly competent *E. coli* could be helpful when IFPC is performed under suboptimal conditions, e.g. when bacteria containing the template vector are used as template, when T4-pnk is employed for phosphorylation, or when templates are poorly amplified. The fact that IFPC allows template flexibility and requires only minimal amount of starting material renders IFPC a promising cloning strategy for low-copy vectors.

On the other hand, limitations regarding PCR should be kept in mind when IFPC is planned. Since IFPC results in an exponential amplification of the template, it exhibits all advantages and disadvantages of conventional PCRs. The insert and the vector have to be amplifiable and if the efficiency of PCR is low, resulting in small counts of colonies, 5 or more PCR cycles should be added to the inverse fusion PCR program, while introduction of more template DNA will result in higher numbers of background colonies. One example for poor template amplification resulting in reduced colony yield is the *lacZ* sequence present as *lacZα* fragment in many cloning vectors such as pCR2.1 or as full *lacZ* in the vector pBAD-TOPO/*lacZ*/V5-His. The low yield of colonies could be increased successfully after adding more cycles and this showed that for some IFPCs optimization of the inverse fusion PCR is necessary. Due to primer mismatching, repeated sequences, or GC-rich domains, the inverse fusion PCR can fail, thus resulting in low numbers of positive colonies and/or a high background of false positive clones. As recommended for the used Phusion DNA polymerase, the annealing temperature can be raised to allow for a more specific primer binding. In any case the insertion sites and primers should be chosen with care, and for IFPC on new vectors a pilot experiment with different primer combinations is recommended. Moreover, multiple or serial insertions by IFPC could be prone to PCR artefacts, since for each insertion, the vector undergoes 25 and the insert 50 PCR cycles. The estimated percentage of PCR products having an error can be calculated for Phusion polymerase (http://www.finnzymes.fi/pcr/fidelity_calc.php) and ranges on 50 cycles between 2.2% for a 1 kb insert and 6.6% for a 3 kb insert and on 25 cycles for a 5 kb vector at 5.5%. Therefore, especially for smaller inserts the risk of PCR derived artefacts is negligible, but if the plasmid backbone contains functional elements, a confirmation by sequencing might be necessary. At last, IFPC may not be suitable for the cloning of cDNA libraries since the inverse fusion PCR step exponentially amplifies some initial vector/insert fusion events and may additionally select inserts as a function of their sizes and GC contents; thus, the resulting libraries will be biased. Therefore, IFPC is best suited for the cloning of single sequences. The assemblage of multiple inserts containing homologous overhangs as shown for CPEC [12] should be possible by IFPC but was not tested yet.

To mimic a standard cloning with a non selectable insert the failure frequency of IFPC was analyzed employing a column-cleaned PCR product and only 6.5% of the colonies contained a non functional insertion. Half of the failure events could be decreased by employing gel-eluted insert DNA, because fusions between the template vector and genomic bacterial DNA were detected. Interestingly, the other half of the failed fusions contained C-terminal truncated sequences and no mismatching events on the insertion boundaries, showing that the overlap extension part of the inverse fusion PCR worked perfect in this setup.

The number of background colonies containing the template vector without insert is constantly very low, up to 50 or less cfu. This is a result of using a minimal starting amount of circular template vector. It is possible to reduce the background by Dpn1 digestion, but based on the low occurrence and the high positive clone counts, this is not necessary. IFPC is suitable for a variety of vectors. Until now, inserts between 100 bp up to 3.5 kb have been successfully cloned into the vectors pBAD, pBAD/Thio, pET-151, pCR2.1 (all Invitrogen), pMK90 [19], pSex81 [20] or pWPI (Addgene plasmid 12254) to insert genes such as *gfp*, produce gene fusions, swap domains or exchange antibiotic resistances. Other vector origins than pUC or pBR322 were not tested until now, but the vector origin should not play a role for successful cloning if it is amplifiable by PCR.

IFPC can be carried out directly with bacteria containing the template vector and insert from a diluted PCR, avoiding any DNA cleanup or digestion step during the whole procedure, thus with only minimal experimental handling. In conclusion, IFPC is a robust and user friendly method for the directional and seamless cloning in *E. coli*.

## Materials and Methods

### IFPC working protocol

To achieve most colonies with lowest background, the gel-eluted insert was used containing a 5′ vector complementary end with an annealing Tm of around 58°C (5′-end of primer A). The inverse fusion PCR was prepared by mixing 500 nM primer B and C, 10 pM circular template vector and 1 nM of the gel-eluted insert, corresponding to a molar ratio V to I of 1: 100. For 1 µl of inverse fusion PCR mix, approximately 5 pg kb^−1^ circular template vector and 500 pg kb^−1^ insert DNA was used. The inverse fusion PCR was run by using Phusion DNA-polymerase (0.02 units µl^−1^) under the following conditions: 98°C for 3 min, 25 cycles of 98°C – 20 s, 58°C – 30 s, 72°C – 30 s kb^−1^ and a final extension step at 72°C for 7 min. The 72°C elongation step was calculated by adding the size of the vector and the insert, and then 2 µl of the inverse fusion PCR was mixed with 2.5 µl Quick or Rapid 2× ligation reaction buffer and subsequently 0.5 µl T4-DNA ligase was added and incubated for 15 min at RT prior to transformation into competent *E. coli* cells. If non phosphorylated primers were used for the inverse fusion PCR, the amount of inverse fusion PCR was reduced and filled up to 2 µl with H_2_O. 2.5 µl Quick or Rapid 2× ligation reaction buffer, 5 units of T4-polynucleotid kinase and 0.5 µl T4-DNA ligase were added into the ligation mix and incubated for 30 min at RT prior transformation.

Troubleshooting: once problems concerning buffers, enzymes or competent cells were eliminated, two additional problems occurred: (i) unspecific primer binding was eliminated by designing new primers; (ii) poor amplification during the inverse fusion PCR was overcome by the addition of cycles (e.g. for vectors containing *LacZ* sequences). In some cases PCR optimization by adding GC-buffer (FinnZymes/NEB), DMSO or OneTaq High GC Enhancer (NEB) was necessary. Even if the inverse fusion PCR worked perfectly, only a very small amount of product was amplified, which was almost not visible on an agarose gel: the absence of a visible product was not indicative for the failure of the inverse fusion PCR reaction.

### Setup and evaluation of IFPC

To change the ampicillin resistance of the vector pBAD (Invitrogen) to a kanamycin resistance, two PCRs and one ligation were performed. For all PCRs, the high-fidelity Phusion DNA Polymerase (0.02 units µl^−1^), HF-buffer (both Finnzymes) and 500 nM primers (desalted, Sigma) were used. All primer sequences are listed in Table 2. To prepare the insert, the coding sequence for kanamycin was amplified in a first PCR employing primer A1 and primer B1, using 1 ng of the vector pCR2.1 (Invitrogen) as template. The vector-homologous part of primer A1 had a calculated annealing Tm of around 58°C. After an initial denaturation step at 98°C for 3 min, followed by 25 cycles of 98°C – 15 s, 58°C – 20 s and 72°C – 30 s, a final extension step (7 min) at 72°C was performed. Depending on which approach was used, the resulting PCR product was (i) diluted in H_2_O, (ii) column cleaned or (iii) gel-eluted using the high pure PCR product purification kit (Roche).

**Table 2:** Oligonucleotides.

project	primer	Sequence (vector-overlap in bold)
kanamycin	Primer A1 (bad-kan-dw)	5′ - **CTT CAA TAA TAT TGA AAA AGG AAG AGT** ATG ATT GAA CAA GAT GGA TTG CAC G - 3′
	Primer B1 (kan-up)	5′ - TCA ATT CAG AAG AAC TCG TCA AGA AG - 3′
	Primer C1 (P-bad-dw)	5′ - Phospho - CTG TCA GAC CAA GTT TAC - 3′
	Primer C1-b (P-bad-dw-b)	5′ - Phospho - CTG TCA GAC CAA GTT TAC TCA TAT ATA CTT TAG - 3′
	Primer B2 (P-kan-up)	5′ - Phospho - TCA ATT CAG AAG AAC TCG TCA AGA AG - 3′
	Primer C2 (bad-dw)	5′ - CTG TCA GAC CAA GTT TAC - 3′
spectinomycin	Primer A3 (kana-spec-dw)	5′ - **ATT GAA CAA GAT GGA TTG CAC** AGG GAA GCG GTG ATC G - 3′
	Primer B3 (spec-up)	5′ - TTT GCC GAC TAC CTT GG - 3′
	Primer C3 (P-dw)	5′ - Phospho - TAT CGC CTT CTT GAC GAG - 3′
	Sequencing (k-dw)	5′ - GCA AAG TAA ACT GGA TGG - 3′
	Sequencing (a-up)	5′ - CAC CCA ACT GAC TTC AG - 3′

Subsequently, a second PCR, the inverse fusion PCR, was performed in order to fuse the kanamycin sequence to the template vector pBAD (Invitrogen). To identify the best conditions, different amounts (1 pg to 50 ng) of circular pBAD and the kanamycin insert as gel-eluted from the first PCR (molar ratio from 1: 0.1 to 1: 1,000) were mixed with a 5′-phosphorylated vector primer C1 and the insert primer B1, buffer and polymerase as above in a total volume of 20 µl. The PCR was then performed with the following profile: 98°C for 3 min, followed by 25 cycles of 98°C – 20 s, 65°C – 1 s, slowly decreasing at 0.1°C s^−1^ to 58°C, 58°C – 30 s, 72°C - 3 min and a final step at 72°C for 10 min. For some settings only one short annealing step (30 s at 58°C) or a non-phosphorylated primer C2 and a phosphorylated primer B2 were used.

For the ligations, 2 µl of the resulting fusion PCRs were mixed with 2.5 µl 2× Quick ligation buffer and 0.5 µl Quick ligase (both NEB) to prepare 5 µl ligation reactions. Alternatively, the rapid ligation system (Promega) was used. The ligation reactions were carried out at room temperature for 15 min, or for 30 min, in the cases where 5 units of T4-polynucleotid kinase (NEB, M0236S) were added to simultaneously phosphorylate the 5′-ends of the inverse fusion products. Finally, the whole reaction was used to transform 50 µl chemically competent *E. coli* Top10 cells (8.5×10^8^ cfu µg^−1^ pUC19) prepared and handled according to the CCMB80 protocol (http://openwetware.org/wiki/TOP10_chemically_competent_cells). To check the insertion of the kanamycin resistance gene, different amounts (1 to 100 µl) of the transformations were plated on LB-Agar containing 50 µg ml^−1^ kanamycin or 100 µg ml^−1^ ampicillin, thus to identify the background of residual pBAD vector.

### Error frequency of IFPC

Since the described setup selected only functional active kanamycin resistant colonies, an additional experiment was performed to identify the percentage of failed fusions. Therefore, a spectinomycin/streptomycin resistance gene (*aadA*, Genbank acc. No. M60473.1) derived from genomic DNA of *E. coli* rl0282 was fused in frame into the kanamycin resistance gene of the vector pCR2.1 (Invitrogen) by simultaneously deleting the kanamycin sequence. The heterologuous primer A3 was designed to bind downstream of the start ATG of *kanamycin*, leading to a *spectinomycin* sequence containing 24 kanamycin derived bases at its 5′-end. This setup should allow the identification of non functional fusions appearing as a result of miss-matches during insert-vector fusion. Primer B3 was chosen to anneal at the 3′-terminus of the kanamycin sequence. With the same settings as described above for the first insert PCR, *aadA* was amplified with the primers A3 and B3, followed by a column purification of the PCR product. For the inverse fusion PCR phosphorylated primer C3, 400 pg µl^−1^ spectinomycin insert and 20 pg µl^−1^ pCR2.1 were used, and PCR was performed under the same conditions as described above, except for a 30 s annealing step at 58°C. The transformed *E. coli* were seeded on ampicillin plates allowing growth of bacteria containing the non-fused vector (amp^R^, kan^R^), the functional fusions (amp^R^, spec^R^) and the non-functional fusions (amp^R^, spec^S^). 192 randomly picked colonies were seeded into wells of 96-well plates containing LB- media with ampicillin (100 µg ml^−1^), kanamycin (50 µg ml^−1^) or spectinomycin (50 µg ml^−1^). 96 of the functional fusion colonies were analyzed by PCR using the primers k-dw and a-up flanking the spectinomycin insertion site in a distance of 116 or 162 bases. The non-functional fusions, growing in the presence of ampicillin but not kanamycin and spectinomycin, were sequenced with the primers k-dw and a-up to identify the reason of fusion failure. In addition, 10 colonies containing the functional fusion were sequenced to confirm the presence of the inserted spectinomycin sequence and its 5′- and 3′- insertion boundaries.

## References

[1] Jung V, Pestka SB, Pestka S (1993). Cloning of polymerase chain reaction-generated DNA containing terminal restriction endonuclease recognition sites.. Methods Enzymol.

[2] Zhou MY, Gomez-Sanchez CE (2000). Universal TA Cloning.. Curr Issues Mol Biol.

[3] Yang J, Zhang Z, Zhang XA, Luo Q (2010). A ligation-independent cloning method using nicking DNA endonuclease.. Biotechniques.

[4] Quinn L (2005). Seamless cloning and gene fusion.. Trends Biotechnol.

[5] Engler C, Gruetzner R, Kandzia R, Marillonnet S (2009). Golden Gate Shuffling: A One-Pot DNA Shuffling Method Based on Type IIs Restriction Enzymes.. PLoS ONE.

[6] Bubeck P, Winkler M, Bautsch W (1993). Rapid cloning by homologous recombination in vivo.. Nucleic Acids Res.

[7] Zhang Y, Werling U, Edelmann W (2012). SLiCE: a novel bacterial cell extract-based DNA cloning method.. Nucleic Acids Research.

[8] Mamie ZL, Elledge SJ (2007). Harnessing homologous recombination *in vitro* to generate recombinant DNA via SLIC.. Nat Methods.

[9] Li C, Wen A, Shen B, Lu J, Huang Y (2011). FastCloning: a highly simplified, purification-free, sequence- and ligation-independent PCR cloning method.. BMC Biotechnology.

[10] Tillett D, Neilan BA (1999). Enzyme-free cloning: a rapid method to clone PCR products independent of vector restriction enzyme sites.. Nucleic Acids Res.

[11] Gibson DG, Young L, Chuang R, Venter JC, Hutchison CA (2009). Enzymatic assembly of DNA molecules up to several hundred kilobases.. Nat Methods.

[12] Quan J, Tian J (2009). Circular Polymerase Extension Cloning of Complex Gene Libraries and Pathways.. PLoS ONE.

[13] Chen GJ, Qiu N, Karrer C, Caspers P, Page MGP (2000). Restriction site free insertion of PCR products directionally into vectors.. Biotechniques.

[14] van den Ent F, Löwe J (2006). RF cloning: a restriction-free method for inserting target genes into plasmids.. J Biochem Biophys Methods.

[15] Bryskin AV, Matsumura I (2010). Overlap extension PCR cloning: a simple and reliable way to create recombinant plasmids.. Biotechniques.

[16] Horton RN, Hunt HD, Ho SN, Pullen JK, Pease LR (1989). Engineering hybrid genes without the use of restriction enzymes: gene splicing by overlap extension.. Gene.

[17] Ochman H, Gerber AS, Hartl DL (1988). Genetic applications of an inversed polymerase chain reaction.. Genetics.

[18] Hoskins RA, Stapleton M, George RA, Yu C, Wan KH (2005). Rapid and efficient cDNA library screening by self-ligation of inverse PCR products (SLIP).. Nucleic Acids Res.

[19] Casali N, Konieczny M, Schmidt A, Riley LW (2002). Invasion Activity of a *Mycobacterium tuberculosis* Peptide Presented by the *Escherichia coli* AIDA Autotransporter.. Infect Immun.

[20] Breitling F, Dübel S, Seehaus T, Klewinghaus I, Little M (1991). A surface expression vector for antibody screening.. Gene.

